# Melatonin alleviates neuroinflammation in ischemic stroke by regulating cyclic GMP-AMP synthase– mediated microglial pyroptosis signaling

**DOI:** 10.4103/NRR.NRR-D-24-01070

**Published:** 2025-06-19

**Authors:** Qian Li, Lin Feng, Yu Tian, Erliang Guo, Yiran Li, Jingyan Niu, Haodong Pan, Chun Dang, Yaoheng Lu, Lihua Wang

**Affiliations:** 1 Department of Neurology, the Second Affiliated Hospital of Harbin Medical University, Harbin, Heilongjiang Province, China; 2 Department of Geriatrics, Nanjing Drum Tower Hospital, the Affiliated Hospital of Nanjing University Medical School, Nanjing, Jiangsu Province, China; 3 Department of Thoracic Surgery, Harbin Medical University Cancer Hospital, Harbin, Heilongjiang Province, China; 4 State Key Laboratory of Analytical Chemistry for Life Science, School of Chemistry and Chemical Engineering, Nanjing University, Nanjing, Jiangsu Province, China; 5 Department of Periodical Press, West China Hospital, Sichuan University, Chengdu, Sichuan Province, China; 6 Department of General Surgery, Chengdu Integrated Traditional Chinese Medicine and Western Medicine Hospital, Chengdu, Sichuan Province, China

**Keywords:** cGAS, immune injury, inflammation, ischemic stroke, melatonin, pyroptosis, STING, microglia

## Abstract

Inflammation plays a key role in driving the secondary brain injury that follows ischemic stroke. Melatonin is an endogenous neuroendocrine hormone that regulates mitochondrial homeostasis. However, the role and mechanisms by which melatonin regulates microglial pyroptosis and the inflammatory cascade through double-stranded DNA (dsDNA)-sensing cyclic GMP-AMP synthase (cGAS) signaling warrant further study. Using middle cerebral artery occlusion mice, we investigated the effects of melatonin on cGAS-mediated pyroptosis and neuroinflammation. Middle cerebral artery occlusion model mice exhibited significantly increased DNA damage and cytoplasmic dsDNA release, as reflected by γH2AX staining, as well as heightened activation of the cytosolic dsDNA-sensing cGAS-STING pathway, both of which were notably suppressed by melatonin treatment. Melatonin also mitigated NOD-like receptor family pyrin domain-containing protein 3 (NLRP3) inflammasome activation and nuclear factor (NF)-κB/gasdermin D-mediated pyroptosis in microglia following ischemic stroke, while exhibiting the capacity to attenuate the immune response to ischemia in mice. This led to reduced infiltration of peripheral neutrophils and monocytes/macrophages in the ischemic brain. Specifically, melatonin administration resulted in reductions in the numbers of ionized calcium-binding adapter molecule 1-positive cells and production of interleukin-6 and tumor necrosis factor-α by microglia. Regarding neurological outcomes, melatonin significantly reduced cerebral infarct volume and ameliorated neurological deficits in mice. Notably, the neuroprotective effect of melatonin was correlated with the inhibition of cGAS activity. We also developed and tested melatonin co-loaded macrophage membrane-biomimetic reactive oxygen species-responsive nanoparticles (Mф-MLT@FNGs), which exhibited therapeutic properties in middle cerebral artery occlusion mice. Our findings suggest that melatonin acts on microglial pyroptosis to inhibit neuroinflammation and reshape the immune microenvironment through regulation of the cGAS-STING-NF-κB signaling pathway. By doing so, melatonin rescues damaged brain tissue and protects neurological function, highlighting its potential as a neuroprotective treatment for ischemic stroke.

## Graphical abstract

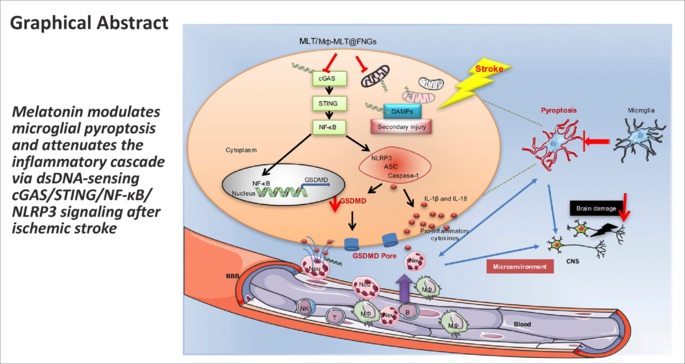

## Introduction

Ischemic stroke is a major disease that threatens human health and survival with high mortality and disability worldwide (Wang et al., 2017; GBD 2019 Stroke Collaborators, 2021). Current first-line treatments, including intravenous thrombolysis and endovascular therapy, aim to restore cerebral blood flow but are limited by a narrow therapeutic window and the risk of hemorrhagic transformation (Yeo et al., 2013). Inflammation plays a central role in driving secondary injury after cerebral ischemia and persists throughout the course of ischemic stroke (Zhou et al., 2018; Iadecola et al., 2020; Huang et al., 2024; Zheng et al., 2025), offering a broad therapeutic window. Therefore, preventing post-stroke inflammation and its downstream effects are crucial for improving ischemic stroke outcomes.

Microglia, as the primary immune defenders in the central nervous system, play a crucial role in ischemic stroke. Activation of microglia and the resulting neuroinflammation are key drivers of secondary brain injury following ischemic events (Lambertsen et al., 2019). Pyroptosis, a form of programmed cell death with inflammatory properties, is characterized by the release of cellular contents through pore formation and is closely linked to ischemic stroke (Yu et al., 2021). This process exacerbates immune damage in cerebral ischemia and is regulated by gasdermin D (GSDMD). Upon cleavage by caspase-1, GSDMD generates its pore-forming active N-terminal fragment (N-GSDMD), leading to cell membrane rupture and the release of inflammatory cytokines, such as interleukin (IL)-18 and IL-1β. These cytokines trigger a robust inflammatory response by recruiting immune cells to the site of brain damage (Long et al., 2023).

Following ischemic stroke, ischemia–reperfusion injury induces severe oxidative stress and energy depletion in brain cells, leading to mitochondrial dysfunction (Qin et al., 2022). Consequently, double-stranded DNA (dsDNA) from damaged nuclei and mitochondria accumulates in the cytoplasm, where it acts as a “danger signal” (Flavell and Sefik, 2024) that can activate the cyclic GMP-AMP synthase (cGAS)-stimulator of interferon genes (STING) pathway. This key pathway drives inflammation by promoting the production of type I interferon (Wan et al., 2020) and triggering the production of pro-inflammatory cytokines via nuclear factor kappa-B (NF-κB) transcription factor (Sun et al., 2013), thus establishing an effective innate immune response. Furthermore, the cGAS-STING pathway acts as a pivotal mediator of inflammation in contexts of infection, cellular stress, and tissue damage (Hopfner and Hornung, 2020; Decout et al., 2021), and is also reportedly involved in the pyroptotic process (Liu et al., 2024; Peng et al., 2024). Inhibition of dsDNA-sensing cGAS can reduce inflammatory responses and offers therapeutic potential for various diseases, such as age-related macular degeneration, ischemic stroke (Kerur et al., 2018; Li et al., 2020; Zhang et al., 2022), Alzheimer’s disease (Xie et al., 2023), ocular surface inflammation (Ouyang et al., 2023), doxorubicin-induced cardiotoxicity (Luo et al., 2023), and prostatitis (Chen et al., 2024)

Melatonin, a hormone secreted by the pineal gland, is a versatile compound with antioxidant and anti-inflammatory properties (Hardeland, 2019). Previous studies have shown that melatonin exerts neuroprotective effects through a variety of mechanisms, including maintaining mitochondrial homeostasis, and inhibiting oxidative stress, inflammatory responses, and cell death (Alghamdi, 2018; Arioz et al., 2019), which may lower the release of dsDNA into the cytoplasm. Melatonin reportedly controls inflammatory gene expression by regulating the NF-κB, hypoxia-inducible factor, nuclear factor erythroid 2-related factor 2, and other signaling pathways, while affecting the mitogen-activated protein kinase cascade reaction, thus comprehensively inhibiting the inflammatory response (Mauriz et al., 2013; Zhao et al., 2015). Despite its known anti-inflammatory benefits, the potential regulatory role and underlying mechanism of melatonin in microglial pyroptosis via the dsDNA-sensing cGAS-STING pathway remain unclear, warranting further investigation.

In this study, we aimed to elucidate the involvement and mechanism of melatonin in alleviating neuroinflammation and brain injury in ischemic stroke through modulation of the cGAS-mediated microglial pyroptosis and inflammatory cascades, providing new insights into its therapeutic potential.

## Methods

### Animals

Experiments were performed on male C57BL/6 mice, microglial cGAS conditional knockout (cGAS-KO) mice and wild-type (WT) mice aged 6–8 weeks. C57BL/6 mice (*n* = 196, bodyweight 23–25 g, specific-pathogen-free grade) were purchased from the Second Affiliated Hospital of Harbin Medical University (Heilongjiang, Harbin, China, License No. SCXK (Hei) 2019-001). The cGAS^flox/flox^ mice (Model Organisms Center, Inc., Shanghai, China; Cat# NM-CKO-2111644, License No. SCXK (Hu) 2019-0002) were mated with transgenic mice expressing CX3CR1 promoter-driven Cre recombinase (Model Organisms Center, Inc.; Cat# NM-KI-200157, License No. SCXK (Hu) 2019-0002) to generate mice that lack cGAS expression in microglia (hereafter referred to as cGAS-KO). The mice were treated with tamoxifen for 5 days to activate the recombinase to remove essential loxP-flanked exons. The cGAS^flox/flox^ littermates without the Cre transgene were used as WT controls throughout the study. Because estrogen is a potent neuroprotective factor, we only used male mice in this study, thereby eliminating potential influences from menstruation-related hormonal changes (Sommer, 2017; Vahidinia et al., 2020). All experiments used naïve mice, housed five per cage in a temperature-controlled mouse room (temperature: 24–25°C; humidity: 55%) at the Animal Center of the Second Affiliated Hospital of Harbin Medical University, under the pathogen-free conditions of the vivarium facilities with a standardized light–dark cycle and free access to food and water, without prior drug testing. All experiments and animal care methods were approved by the Animal Ethics Committee of the Second Affiliated Hospital of Harbin Medical University (approval No. 2024GZRYS-226) on February 20, 2024, and were performed in accordance with the National Institutes of Health Guide for the Care and Use of Laboratory Animals (8^th^ ed., National Research Council, 2011) and the ARRIVE 2.0 guidelines (Animal Research: Reporting of *In Vivo* Experiments) (Percie du Sert et al., 2020). Mice were randomly divided into the following treatment groups: sham surgery with vehicle (0.5% dimethyl sulfoxide in saline) control (Sham + Vehicle); sham with melatonin (Sham + MLT); middle cerebral artery occlusion (MCAO) modeling with vehicle (MCAO + Vehicle); MCAO + MLT; and MCAO with melatonin co-loaded macrophage membrane-biomimetic reactive oxygen species (ROS)-responsive nanoparticles (MCAO + Mф-MLT@FNGs) (*n* = 6 mice/group). Body temperature was maintained at 37.0 ± 0.5°C during MCAO surgery, after which the mice were moved to a well-ventilated recovery room maintained at 25 ± 3°C, where they were kept in individual cages and provided with food and water until they regained consciousness. All surgeries were performed with animals under anesthesia, for tissue preparation, mice were anesthetized with intraperitoneal injection of 50 mg/kg pentobarbital sodium (Sigma-Aldrich, Shanghai, China; Cat# P3761). For postoperative analgesia, we used 0.03 mg/kg buprenorphine (Sigma-Aldrich, St. Louis, MO, USA), administered by intraperitoneal injection every 12 hours for 24 hours. All efforts were made to minimize animal suffering and limit the number of animals used.

### Middle cerebral artery occlusion modeling

Focal cerebral ischemia was induced via occlusion of the left middle cerebral artery to create MCAO model mice. Briefly, mice were anesthetized by inhalation with 3% isoflurane (R510-22-10; RWD, Shenzhen, China); anesthesia was maintained with 1.0%–2.0% isoflurane in 70% N_2_O and 30% O_2_. As previously described (Li et al., 2017), the neck skin was exposed under a dissecting microscope, disinfected, and a longitudinal incision was made. The left common carotid artery (CCA), internal carotid artery (ICA), and external carotid artery (ECA) were carefully separated, the left CCA and ECA were ligated, a small incision was made at the distal CCA, and a standardized silicone-coated nylon thread (701956PK5Re, Doccol, Sharon, MA, USA) was inserted into the left ICA (8–9 mm) through the CCA incision to occlude the middle cerebral artery. After 60 minutes, reperfusion was established by gently withdrawing the tether to the CCA. Sham mice were subjected to the same experimental procedure, but the tether was withdrawn immediately after reaching the middle cerebral artery. To ascertain the success of arterial occlusion, cerebral blood flow (CBF) was measured in the left frontoparietal cortical region using a laser Doppler flowmeter (PF5010, PeriFlux 5000, Perimed, Stockholm, Sweden). Only MCAO mice with a > 85% reduction in baseline (pre-ischemic) CBF during ischemia and a > 80% increase in baseline CBF during reperfusion were included in the study. During the procedure, body temperature was maintained using an electric heating blanket. Melatonin (Sigma-Aldrich) was dissolved in dimethyl sulfoxide and diluted in normal saline solution to a final concentration of < 2% dimethyl sulfoxide (vehicle). Melatonin or Mф-MLT@FNGs were intravenously injected into the tail veins of mice at a dose of 10 mg/kg, which was chosen on the basis of previous studies (Yang et al., 2015; Xu et al., 2018; Zhang et al., 2018), at 30 minutes before MCAO, 30 minutes post reperfusion, and then once daily for 3 days post reperfusion.

### Neurobehavioral test

At the indicated post-MCAO time points, two investigators who were blinded to the group assignments assessed the neurological function of mice using a battery of tests which included the modified Neurological Severity Score (mNSS) and the corner turning test (Clarkson et al., 2010; Li et al., 2019; Ren et al., 2023). The mNSS ranges from 0 to 18, with scores interpreted as follows: 13–18 represents severe injury; 7–12 represents moderate injury; and 1–6 represents mild injury. For the corner test, mice were allowed to enter a 30° corner with exits available on both the left and right sides, and the score was calculated as the percentage of left turns out of a total of 10 trials: (number of left turns/10 trials) × 100%.

### 2,3,5-Triphenyltetrazolium chloride staining

On day 3 post reperfusion, brains were sectioned into 2-mm-thick coronal sections using a mouse brain microtome (Zivic Instruments, Pittsburgh, PA, USA), then incubated in 2.0% 2,3,5-triphenyltetrazolium chloride (TTC) (Sigma) at 37°C for 20 minutes. Stained sections were then fixed in 4% paraformaldehyde (PFA) and photographed 30 minutes later with a digital camera. Infarcted areas (white sections) in each section were assessed quantitatively using ImageJ software (version 1.53a, National Institutes of Health, Bethesda, MD, USA).

### Immunohistochemical staining

At 3 days post-reperfusion, mice were anesthetized and intraventricularly perfused with chilled phosphate-buffered saline (PBS). Brain tissues were removed and fixed by immersion in 4% PFA overnight, followed by successive concentrations of 15% and 30% sucrose at 4°C overnight. The tissues were embedded with optimal cutting temperature compound, and 8-μm coronal pathological sections were prepared using a cryostat microtome (Leica Microsystems LM3050S, Wetzlar, Germany) and stored in a –80°C freezer. For immunohistochemistry, frozen sections (8 μm) were incubated in 0.3% hydrogen peroxide for 20 minutes to inactivate endogenous peroxidases, as previously described (Li et al., 2020). After washing in PBS, brain sections were blocked in 5% bovine serum albumin (BSA) for 1 hour at room temperature. The slides were incubated overnight at 4°C with the following primary antibodies: goat anti-IL-1β (1:200; R and D Systems, Minneapolis, MN, USA, Cat# MAB401, RRID: AB_2124620); mouse anti-caspase-1 (1:200, BioLegend, San Diego, CA, USA, Cat# 645102, RRID: AB_2068900); and mouse anti-GSDMD (1:100, Santa Cruz Biotechnology, Dallas, TX, USA, Cat# sc-393581, RRID: AB_2819179). Using biotinylated secondary antibodies, the Vectastain Avidin-Biotin Complex (ABC) kit and a 3,3′-diaminobenzidine tetrachloride (DAB) peroxidase substrate kit (both from Vector Laboratories, Burlingame, CA, USA) were employed to detect immunoreactivity. All slides were imaged using an Axioskop2 microscope (Zeiss MicroImaging Inc., Baden-Württemberg, Germany). A set of sections that were stained similarly but without the primary antibody served as the negative control.

### Immunofluorescence assay

Immunofluorescence analysis was performed as previously described (Li et al., 2020). Briefly, frozen coronal brain sections were treated as indicated, fixed with 4% PFA, and then blocked with 5% BSA in PBS for 1 hour at room temperature. For immunofluorescent staining, sections were incubated overnight at 4°C with the following antibodies: rabbit anti-γH2AX (1:100, Novus Biologicals, Littleton, CO, USA, Cat# NB 100-384, RRID: AB_350295); rabbit anti-ionized calcium-binding adapter molecule 1 (Iba-1; 1:500, FUJIFILM Wako Pure Chemical Corporation, Osaka, Japan, Cat# 019-19741, RRID: AB_839504); and mouse anti-GSDMD (1:100, Santa Cruz Biotechnology, Cat# sc-393581, RRID: AB_2819179). The next day, the sections were washed with ice-cold PBS, and then incubated with one of the following secondary fluorescent-conjugated antibodies for 1 hour at room temperature in the dark: goat anti-rabbit Alexa Fluor 594 (1:500, Thermo Fisher Scientific, Waltham, MA, USA, Cat# A-11012, RRID: AB_2534079); goat anti-rabbit Alexa Fluor 488 (1:500, Thermo Fisher Scientific, Cat# A-11008, RRID: AB_143165); or goat anti-mouse Alexa Fluor 594 (1:500, Thermo Fisher Scientific, Cat# A-11005, RRID: AB_2534073). DAPI (Abcam, Cambridge, MA, USA) was used for nuclear staining. Slides were observed and photographed using a confocal microscope (Olympus, Heidelberg, Germany). Image analysis was performed using ImageJ software (version 1.53a). Regions of interest (ROIs) were selected using consistent thresholding, and the mean fluorescence intensity (MFI) was quantified.

### Flow cytometry

At 3 days post MCAO, cerebral tissues were harvested and perfused with cold PBS, as previously described (Ren et al., 2018), followed by gentle mechanical homogenization using 40-μm nylon cell strainers (Becton Dickinson, Franklin Lakes, NJ, USA) in PBS on ice. After centrifugation, the cell pellets were collected, resuspended in 5 mL of 30% Percoll (GE Healthcare Bio Science AB, Uppsala, Sweden), pelleted again at 700 × *g* for 10 minutes, harvested, and washed once with 5 mL 1% BSA solution in preparation for staining. Following the manufacturer’s protocols, the freshly obtained brain cells were immunostained with mouse-reactive BioLegend antibodies against the following factors and directly labeled with fluorescein isothiocyanate (FITC), phycoerythrin (PE), allophycocyanin (APC), or PerCP-Cy5.5CD45 (30-F11): CD11b (M1/70), CD3 (145-2C11), CD4 (GK1.4), CD8 (53-6.72), NK1.1 (PK136), CD19 (1D3), F4/80 (6F12), Ly6G (1A8), IL-6 (MP5-20F3), tumor necrosis factor (TNF)-α (MP6-XT22), IL-4, and transforming growth factor (TGF)-β (TW7-20B9). Antibody staining was performed in accordance with the manufacturer’s instructions, including additional cell fixation and permeabilization processes needed for staining of intracellular antigens. Fluorescence minus one (FMO) controls were stained at the same time. Data on cell-surface phenotype and intracellular cytokine expression were collected using a FACS Aria III (BD Bioscience, San Jose, CA, USA), and data were analyzed by FlowJo version 10 (flowjo.com).

### Western blotting

Total protein was extracted from lysed brain tissues. Following the determination of protein concentration using a bicinchoninic acid (BCA) kit (Beyotime, Shanghai, China), equal amounts of protein were separated by sodium dodecyl sulfate (SDS)-polyacrylamide gel electrophoresis and transferred to polyvinylidene fluoride membranes (Immobilon-P Transfer Membrane, Millipore, Billerica, MA, USA). The membranes were blocked with 5% BSA in Tris-buffered saline containing 0.1% Tween-20, then incubated with the following specific primary antibodies at 4°C overnight: rabbit anti-cGAS (1:1000, Cell Signaling Technology, Danvers, MA, USA, Cat# 31659, RRID: AB_2799008); rabbit anti-STING (1:1000, Cell Signaling Technology, Cat# 13647, RRID: AB_2732796); rabbit anti-NF-κB (1:1000, Cell Signaling Technology, Cat# 8242, RRID: AB_10859369); rabbit anti-NOD-like receptor family pyrin domain-containing protein 3 (NLRP3; 1:1000, Cell Signaling Technology, Cat# 15101, RRID: AB_2722591); rabbit anti-caspase-1 (1:1000, Cell Signaling Technology, Cat# 24232, RRID: AB_2890194); mouse anti-IL-1β (1:1000; Cell Signaling Technology; Cat# 57058); rabbit anti-IL-18 (1:1000, Cell Signaling Technology, Cat# 57058); rabbit polyclonal anti-β-actin (1:2500, Proteintech, Chicago, IL, USA, Cat# 20536-1-AP, RRID: AB_10700003). The next day, the membranes were washed and incubated with one of the following secondary antibodies at room temperature for 1–2 hours: goat anti-rabbit IgG (1:5000, ABclone, Boston, MA, USA, Cat# AS014, RRID: AB_2769854) or goat anti-mouse IgG (1:5000, ABclone, Cat# AS062, RRID: AB_2864056). Immunoreactive bands were detected using an enhanced chemiluminescence reagent (ECL) kit (Vazyme, Nanjing, China). The intensity of each band area was quantified using ImageJ (version 1.53a).

### Cell culture and treatment

BV2 cells were obtained from Servicebio (Wuhan, Hubei, China; Cat# STCC20009G-1) and cultivated in Dulbecco’s modified Eagle’s medium (DMEM; Gibco, Carlsbad, CA, USA) supplemented with 10% fetal bovine serum (FBS; Gibco), and 100 U/mL penicillin and 100 mg/mL streptomycin (Gibco) at 37°C in a humidified cell incubator. For stimulation, BV2 cells were treated with 100 ng/mL lipopolysaccharide derived from Escherichia coli O55:B5 (Sigma-Aldrich) for 12 hours.

### Motif enrichment analysis and chromatin co-immunoprecipitation assay

The JASPAR database of transcription factor binding profiles (https://jaspar.genereg.net/) was utilized to predict potential transcription factor binding sites in the promoter regions of target genes. Promoter sequences were retrieved, and motif enrichment analysis was conducted using the JASPAR CORE collection. Chromatin co-immunoprecipitation (ChIP) assays were performed following the instructions of a ChIP Assay Kit (Beyotime, Cat# P2078). Briefly, BV2 cells were treated with 100 ng/mL lipopolysaccharide for 12 hours, passaged, and collected when the cell density reached 90%. A 10-mL volume of fresh complete medium and 225 μL 37% PFA were added to the cells, mixed gently, and incubated at room temperature for 10 minutes to lyse the cells for crosslinking. To terminate the crosslinking, 1 mL 10 × glycine solution was added to the dish, mixed well, and incubated at room temperature for 5 minutes. After washing with PBS, the supernatant was discarded, 10 mL pre-cooled PBS containing 1 mM Protease Inhibitor Cocktail II was added, and the cells were transferred to new 1.5-mL Eppendorf tubes. After collecting the cells by centrifugation at 1500 × *g* for 2 minutes in a precooled centrifuge, the supernatant was discarded. The cells were resuspended in 1 mL SDS mixed with 5 μL Protease Inhibitor Cocktail II to prepare a lysate. Cellular DNA was fragmented using a sonicator, followed by centrifugation at 12,000 × *g* for 10 minutes at 4°C to collect the supernatant. Sonication products were incubated on ice with 900 μL ChIP dilution buffer and 60 μL Protein G Agarose for 1 hour at 4°C, and centrifuged at 10,000 × *g* for 1 minute at 4°C. A 10-μL aliquot of the supernatant was pipetted as Input, and the remaining supernatant was divided equally into three new Eppendorf tubes, to which the corresponding antibody was added for an overnight incubation at 4°C: 1 μg anti-RNA polymerase antibody (positive control); 1 μg anti-rabbit IgG (negative control); or 2 μg anti-NF-κB antibody (experimental group). The following day, 60 μL Protein A+G Agarose was added to each tube, incubated at 4°C for 1 hour, and centrifuged at 10,000 × *g* for 1 minute. The supernatant was discarded, and the pellet was washed prior to elution. Gradually, 1 mL each of low-salt, high-salt, and LiCl immune complex wash buffers was added to the pellet to wash the precipitated DNA/protein complexes. Subsequently, 8 μL of sodium chloride solution was added to the eluent, and the complexes were de-crosslinked in a water bath at 65°C for 4.5 hours. The samples were then processed to remove RNA and purify the DNA, which was then used as template in PCR to detect the presence or absence of binding frames. The GSDMD primers used for the ChIP assay were as follows: forward, 5′-AAT CTG GGG TTG AGG ATG ACC-3′; reverse, 5′-CAC ACA TTC ATG GAG GCA-3′.

### Construction of Mф-MLT@FNGs

A 10 mg quantity of polymeric conjugate comprising cyanine 7 (Cy7), polyethylene glycol (PEG), thioketal (TK), and poly(lactic-co-glycolic acid) (PLGA) (Xi’an ruixi Biological Technology, Shanxi, China, Cat# R-PF-0138) was dissolved in 1 mL dimethylformamide (DMF), added dropwise to 10 mL ddH_2_O over 30 minutes of vigorous stirring, and emulsified by 5 minutes of ultrasonication. The mixture was centrifuged at 3000 × *g* for 20 minutes to remove agglomerates and nanoparticles larger than the desired particle size, followed by ultrafiltration at 4200 × *g* using a 100-kDa ultrafiltration tube and three washes to produce Cy7-PEG-TK-PLGA fluorescent nanogels (FNGs). To prepare melatonin-loaded nanoparticles (MLT@FNGs), 0.1 mg melatonin was dissolved in 1 mL DMF containing 10 mg Cy7-PEG-TK-PLGA. Under vigorous stirring, 10 mL ddH_2_O was added dropwise over 30 minutes followed by emulsification using sonication for 5 minutes, centrifugation at 5000 × *g* for 20 minutes to remove large agglomerates and nanoparticles, and ultrafiltration at 5000 rpm using a 100-kDa ultrafiltration membrane and three washes.

To prepare Mф-MLT@FNGs, the MLT@FNGs were mixed with 1 mL of macrophage membranes extracted from logarithmic-phase RAW264.7 macrophages (Servicebio, Cat# STCC20020G-1) and subjected to back-and-forth extrusion using a 1-mL miniature extruder, followed by 20 back-and-forth extrusions each through 400-, 200-, and 100-nm polycarbonate membranes. The corresponding particle sizes and zeta potentials of the two formulations were successively measured and analyzed using a Zetasizer Nano ZS laser particle sizer (Malvern Panalytical Ltd., Malvern, UK). The morphologies of MLT@FNGs and Mф-MLT@FNGs were examined under transmission electron microscopy (TEM) using a F200X S/TEM (Thermo Fisher Scientific).

### Dynamic light scattering analysis

MLT@FNGs were dissolved in ddH_2_O to prepare a solution of an appropriate concentration, which was subjected to ultrasonic treatment to ensure uniform dispersion. A 2-mL sample was then transferred to the sample chamber for dynamic light scattering (DLS) analysis, and the particle size distribution data were recorded.

### Ultraviolet spectrophotometry of melatonin drug release

A full-spectrum scan of melatonin in a 30% ethanol aqueous solution was performed to determine the maximum absorption wavelength, identified as 278 nm. The absorbances of melatonin solutions at concentrations of 50, 40, 30, 20, and 10 mg/L were measured at this wavelength to establish a standard calibration curve. Subsequently, MLT@FNGs containing 250 µg of melatonin were weighed and dissolved in 5 mL of 0.5 mM hydrogen peroxide (H_2_O_2_). At intervals of 0, 1, 2, 3, and 4 hours, 200 µL of the solution was sampled, and the absorbance at 278 nm was recorded. The cumulative drug release rate was then calculated based on the standard curve.

### Statistical analysis

All data are presented as means ± standard deviation (SD). Student’s *t*-test was used for comparisons between two groups. One-way analysis of variance followed by Tukey’s *post hoc* test, or two-way analysis of variance with multiple comparisons followed by Bonferroni’s *post hoc* test, were used for comparisons of multigroup data in GraphPad Prism (version 9.0, GraphPad, San Diego, CA, USA, www.graphpad.com). A *P* value < 0.05 was considered statistically significant.

## Results

### Cytoplasmic double-stranded DNA is upregulated and melatonin inhibits the cyclic GMP-AMP synthase-STING pathway following brain ischemia

**Figure 1:**
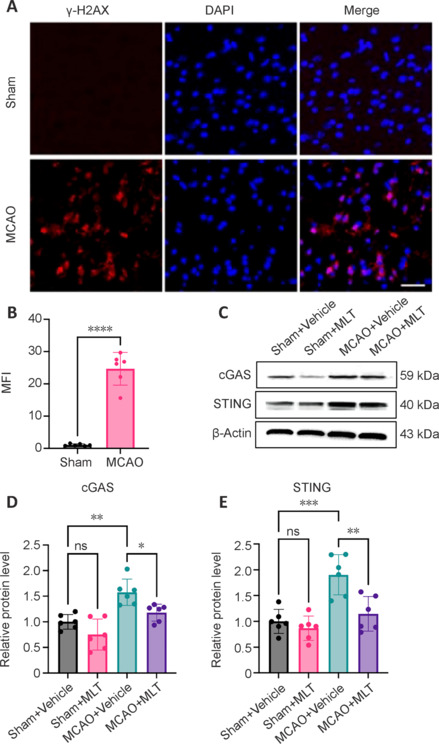
Upregulation of γH2AX and activation of its associated cGAS‒STING pathway following MCAO. (A, B) Immunostaining for γH2AX (red) at 72 hours after MCAO in the peri-ischemic area as well as in the corresponding regions of sham control brains, with summarized results. Nuclei were stained with DAPI (blue). Scale bar: 20 μm. *n* = 6 per group. (C–E) Western blotting for the cGAS-STING pathway at 72 hours after MCAO in ischemic brain tissues and sham controls, both of which were treated with MLT or vehicle, and quantification of the cGAS-STING pathway. The data were normalized to β-actin expression. *n* = 6 per group. Data are presented as mean ± SD. *P < 0.05, **P < 0.01, ***P < 0.001, ****P < 0.0001 (Student’s *t*-test for B; one-way analysis of variance followed by Tukey’s *post hoc* test for D and E). All experiments were conducted in triplicate. cGAS: Cyclic GMP‒AMP synthase; DAPI: 4′,6-diamidino-2-phenylindole; MCAO: middle cerebral artery occlusion; MFI: mean fluorescence intensity; MLT: melatonin; ns: not significant; STING: stimulator of interferon genes.

At 72 hours post MCAO, we assessed DNA damage via immunofluorescence staining of coronal brain sections using γH2AX, a phosphorylated form of histone protein H2A. This analytical approach allowed us to meticulously examine the presence of damaged cells and the release of dsDNA into the cytoplasm (**Figure 1A**), unveiling a substantial upregulation in γH2AX levels within the cytoplasm following ischemic injury (**Figure 1B**). To investigate the capability of melatonin to mitigate this release of dsDNA, we quantified the expression levels of the dsDNA-sensing cGAS-STING pathway using western blotting analysis of brain tissues (**Figure 1C**). At 72 hours post MCAO, mice treated with vehicle control showed robust upregulation of the cGAS-STING pathway, whereas those treated with melatonin showed significant downregulation of this pathway (**Figure 1D** and **E**). These pivotal observations highlighted the acute impact of brain ischemia on DNA integrity while providing evidence that melatonin has the potential to inhibit the cGAS-STING pathway following ischemic stroke.

### Inactivation of microglial cyclic GMP-AMP synthase inhibits the development of pyroptosis following ischemic stroke

**Figure 2:**
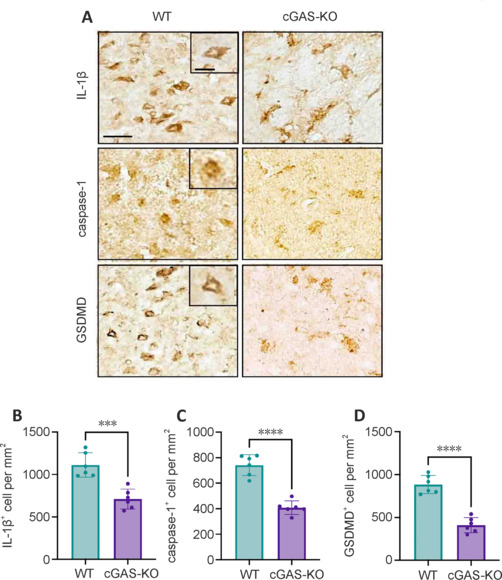
Inactivation of cGAS downregulates the expression of pyroptosis-related proteins following brain ischemia. (A, B) Immunostaining of IL-1β, caspase-1, and GSDMD (brown precipitate) in the peri-infarct area of the brains of WT and cGAS-KO mice at 72 hours following MCAO (A), along with the summarized results (B–D). Scale bars: 20 μm (10 μm in the magnified images). *n* = 6 per group. Data are presented as mean ± SD. ***P < 0.001, ****P < 0.0001 (Student’s *t*-test). All experiments were conducted in triplicate. cGAS: Cyclic GMP-AMP synthase; GSDMD: gasdermin D; IL-1β: interleukin-1β; KO: knockout; MCAO: middle cerebral artery occlusion; WT: wild-type.

To explore the impact of microglial cGAS inactivation on pyroptosis following ischemic stroke, we conducted morphological analyses of brain tissues obtained from WT and microglia-specific cGAS-KO mice using immunohistochemistry. Our focus was on the penumbra area, which represents a pivotal modifiable determinant in ischemic stroke. Histological examination utilizing DAB staining showed that inactivation of microglial cGAS markedly attenuated the levels of activated caspase-1, IL-1β, and GSDMD following MCAO (**Figure 2**). These results suggested that microglial cGAS plays an important role in modulating pyroptotic processes in ischemic stroke, highlighting its potential as a therapeutic target for mitigating ischemia-induced neuroinflammation and brain damage.

### Melatonin reduces NOD-, LRR- and pyrin domain-containing protein 3 inflammasome-associated protein expression and inhibits microglial pyroptosis following ischemic stroke

**Figure 3:**
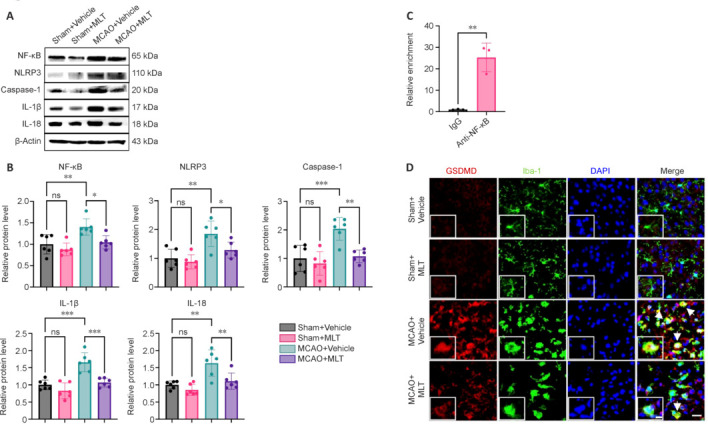
Melatonin suppresses NF-κB/NLRP3 inflammasome activation and microglial pyroptosis in the ischemic brain. (A) Representative western blotting images of NF-κB/NLRP3 inflammasome proteins in ischemic brain tissues and sham controls treated with MLT or vehicle at 72 hours after reperfusion after MCAO. (B) Quantification of the western blotting data. *n* = 6 per group. Data are presented as mean ± SD. *P < 0.05, **P < 0.01, ***P < 0.001 (one-way analysis of variance followed by Tukey’s *post hoc* test). (C) Chromatin immunoprecipitation (ChIP) assay of NF-κB and GSDMD in BV2 cells treated with lipopolysaccharide. *n* = 3 per group. The JASPAR website predicted the existence of a binding site between NF-κB and the GSDMD promoter region (Additional Figure 1). Data are presented as mean ± SD. **P < 0.01 (Student’s *t*-test). (D) Double immunostaining for Iba-1 (green) and GSDMD (red) at 72 hours after MCAO in the peri-ischemic area as well as in the corresponding regions of sham control brains, both of which were treated with MLT or vehicle. Nuclei were stained with DAPI (blue). Scale bar: 20 μm (10 μm in the magnified images). Arrowheads indicate GSDMD-double-positive cells with microglia-specific markers. All experiments were conducted in triplicate. cGAS: Cyclic GMP-AMP synthase; DAPI: 4′,6-diamidino-2-phenylindole; GSDMD: gasdermin D; Iba-1: ionized calcium-binding adapter molecule 1; IL: interleukin; MCAO: middle cerebral artery occlusion; MLT: melatonin; NF-κB: nuclear factor kappa-B; NLRP3: NOD-like receptor family pyrin domain-containing protein 3; ns: not significant.

The cGAS-STING pathway functions as an upstream regulator of the NF-κB pathway, while NF-κB reportedly serves as the priming signal for NLRP3 inflammasome activation. Therefore, we investigated the expression levels of NF-κB, NLRP3, caspase-1, IL-1β, and IL-18 in brain tissues following MCAO. As expected, compared with Sham + Vehicle mice, MCAO + Vehicle mice exhibited significant upregulation of these proteins, which appeared to be reversed by melatonin treatment in the MCAO+MLT group (**Figure 3A** and **B**).

We predicted binding sites for NF-κB on the GSDMD gene promoter using JASPAR (**Additional Figure 1**). Next, a ChIP assay was conducted using BV2, a murine cell line derived from primary microglial cell cultures and immortalized by infection with a v-raf/v-myc oncogene carrying J2 retrovirus. Compared with the IgG control group, there was significant enrichment of NF-κB binding in the GSDMD promoter region in the anti-NF-κB group (**Figure 3C**). Given the pivotal role of microglia in mediating post-stroke inflammation and secondary injury following ischemia, we further investigated whether microglia exhibit heightened GSDMD expression following MCAO. Double immunofluorescence staining demonstrated the co-localization of GSDMD with microglial marker Iba-1 in the peri-ischemic area at 72 hours post MCAO (**Figure 3D**). Compared with the Sham + Vehicle group, a noticeable increase in microglia numbers was observed in the penumbra of the MCAO + Vehicle group, but not the MCAO + MLT group, suggesting that melatonin curtails the proliferation and migration of microglia toward damaged tissue. Additionally, our findings indicated that ischemia substantially heightened pyroptosis, as evidenced by the consistent increase in GSDMD protein levels in the MCAO + Vehicle group, but not in the MCAO + MLT group (**Figure 3D**), suggesting that melatonin effectively counteracted post-stroke microglial pyroptosis. These results underscored the significant role of melatonin in modulating the immune response mediated by cytosolic DNA sensing, providing mechanistic insights into its neuroprotective effects in ischemic stroke.

### Melatonin reduces post-middle cerebral artery occlusion neutrophil/macrophage infiltration, microglial activation, and microglial production of pro-inflammatory factors

**Figure 4:**
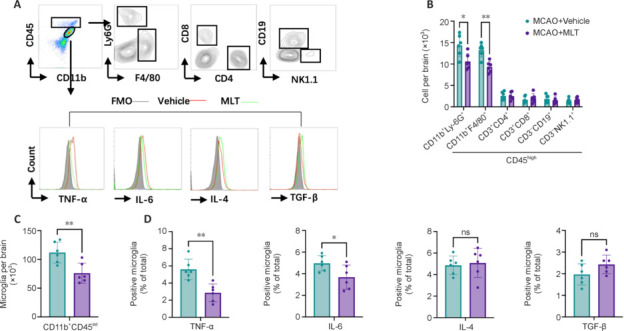
Melatonin reduces microglial activity and alleviates neutrophil/macrophage infiltration and the production of inflammatory factors in MCAO. Groups of mice were administered either melatonin or vehicle on day 3 post-MCAO. (A) Gating strategy for identifying microglia and brain-infiltrating immune cells, including neutrophils (CD45^high^CD11b^+^Ly6G^+^), monocytes/macrophages (CD45^high^CD11b^+^F4/80^+^), CD4^+^ T cells (CD45^high^CD3^+^CD4^+^), CD8^+^ T cells (CD45^high^CD3^+^CD8^+^), B cells (CD45^high^CD3^–^CD19^+^) and NK cells (CD45^high^CD3^–^NK1.1^+^). (B) Counts of brain-infiltrating leukocytes in the indicated groups of mice on day 3 after MCAO. (C) Counts of microglia in the indicated groups of mice on day 3 after MCAO. (D) Production of pro- and anti-inflammatory factors by microglia on day 3 after MCAO. Flow cytometry data depict the expression levels of TNF-α, IL-6, IL-4, and TGF-β1 in the indicated groups of mice. *n* = 6 per group. Data are presented as the mean ± SD. *P < 0.05, **P < 0.01 (Student’s *t*-test). All experiments were conducted in triplicate. FMO: Fluorescence minus one; IL: interleukin; MCAO: middle cerebral artery occlusion; MLT: melatonin; ns: not significant; TGF-β: transforming growth factor-β; TNF-α: tumor necrosis factor-α.

Next, we sought to investigate the impact of melatonin on the immune response following MCAO. Flow cytometry analysis was employed to assess various cellular components in the brains of MCAO mice (**Figure 4A**). Our findings revealed that melatonin treatment led to a significant reduction in the counts of microglia (CD45^int^CD11b^+^) (**Figure 4C**), along with attenuated infiltration of neutrophils (CD11b^+^CD45^high^Ly6G^+^) and monocyte/macrophages (CD11b^+^CD45^high^F4/80^+^) at 72 hours post MCAO compared with vehicle-treated controls (**Figure 4B**). By contrast, no significant differences were observed between melatonin-treated and vehicle-treated mice in the numbers of CD4^+^ T cells (CD45^high^CD3^+^CD4^+^), CD8^+^ T cells (CD45^high^CD3^+^CD8^+^), natural killer (NK) cells (CD45^high^CD3^–^NK1.1^+^), or B cells (CD45^high^CD3^–^CD19^+^). Furthermore, while melatonin treatment resulted in decreased microglia, it also led to reduced microglial expression of TNF-α and IL-6 in MCAO mice (**Figure 4D**). Interestingly, although not statistically significant, melatonin treatment appeared to elevate the microglial expression of TGF-β and IL-4. Collectively, these results underscored the potential of melatonin for attenuating neuroinflammation and mitigating brain inflammatory cascades following MCAO.

### Construction of melatonin co-loaded Mф-MLT@FNGs for enhanced treatment of ischemic stroke

**Figure 5:**
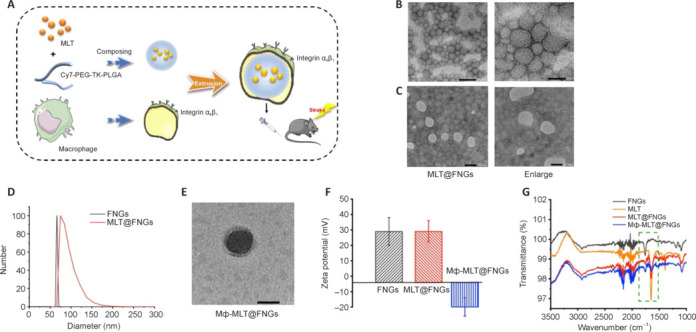
Characterization of Mф-MLT@FNGs. (A) Schematic representation illustrating the preparation process of Mф-MLT@FNGs. (B, C) Transmission electron microscopy images and negative staining images depicting the morphology and structure of the nanogels. Scale bars: 100 nm. (D) Determination of the hydration diameter of the nanogels using dynamic light scattering analysis. (E) Transmission electron microscopy image showing the structure of Mф-MLT@FNGs. Scale bar: 100 nm. (F) Zeta potential measurement. (G) Infrared absorption spectrum analysis of FNG, MLT, MLT@FNGs and Mф-MLT@FNGs. All the experiments were conducted in triplicate. Cy7: Cyanine 7; FNGs: fluorescent nanogels; MLT: melatonin; PEG: polyethylene glycol; PLGA: poly(lactic-co-glycolic acid); TK: thioketal.

To enhance the therapeutic efficacy of melatonin for ischemic stroke, we developed a macrophage membrane biomimetic system involving the synthesis of ROS-responsive Cy7-PEG-TK-PLGA nanoparticles. As illustrated in **Figure 5A**, melatonin was combined with Cy7-PEG-TK-PLGA nanoparticles to form MLT@FNGs, which exhibited uniformity in size, a spherical shape, smooth surface, and tight distribution under TEM and negative staining (**Figure 5B** and **C**). DLS analysis confirmed a hydrated particle size of approximate 75 nm of MLT@FNGs (**Figure 5D**). Ultraviolet absorption features showed a characteristic absorption peak at 277 nm and ROS-responsive melatonin release for MLT@FNGs (**Additional Figure 2**). MLT@FNGs were then fused with cell membranes extracted through extrusion of macrophages to form composite Mф-MLT@FNGs, which were administered to MCAO mice via tail vein injection. TEM images showed that coating with macrophage membranes created Mф-MLT@FNGs exhibiting a complete core-shell structure with a well-defined spherical morphology (**Figure 5E**). Zeta potential assessments indicated strong integration capacity, with an average potential of approximately 30 mV for FNGs and MLT@FNGs. By contrast, the encapsulation of macrophages in Mф-MLT@FNGs resulted in a negative zeta potential of about –20 mV, indicating the formation of new nanogels with composite properties (**Figure 5F**). Infrared absorption spectrum analysis demonstrated the presence of melatonin in the nanoparticles, with characteristic peaks recorded in the range of 3500–1000 nm (**Figure 5G**). Specifically, a peak at **~**1700 cm^–1^ in MLT@FNGs and Mф-MLT@FNGs, indicating the presence of melatonin, was absent in FNGs. These spectra confirmed the encapsulation of melatonin within the artificial membrane assembly of Mф-MLT@FNGs. We also investigated the *in vitro* release of encapsulated melatonin from MLT@FNGs to assess its responsiveness to ROS. In the absence of H_2_O_2_, MLT@FNGs displayed a UV absorption spectrum similar to that of FNGs, indicating minimal melatonin release (**Additional Figure 2A**). However, after incubation with 0.5 mM H_2_O_2_ for 1, 2, 3, or 4 hours, the UV spectrum of MLT@FNGs showed a progressively increasing peak at 278 nm, demonstrating that H_2_O_2_ triggered a rapid release of melatonin from these nanoparticles. Under experimental conditions simulating a high-ROS stroke environment, melatonin release was evaluated over time using UV spectrophotometry. MLT@FNGs exhibited a burst release pattern, with a release rate of 45% within 4 hours (**Additional Figure 2B**). These findings demonstrated that MLT@FNGs possess excellent stability under physiological conditions, respond to ROS in a post-stroke inflammatory environment, and enable the rapid release of melatonin.

### Melatonin and Mф-MLT@FNGs alleviate brain damage and improve neurodeficits following middle cerebral artery occlusion

**Figure 6:**
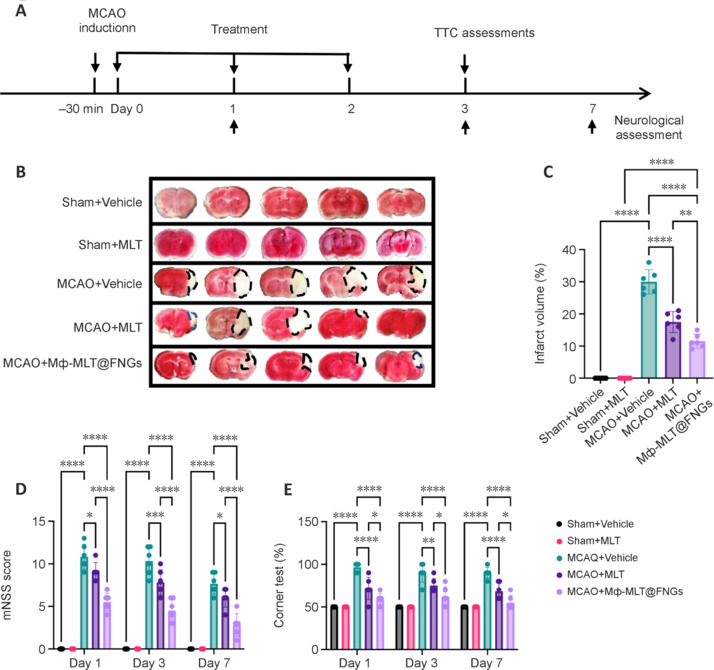
Melatonin and Mф-MLT@FNGs attenuate brain ischemic injury. (A) Flowchart outlining the experimental design. (B, C) Images of 2,3,5-triphenyltetrazolium chloride staining and corresponding quantitative analyses of infarct volume in mice treated with MLT, Mф-MLT@FNGs, or vehicle on day 3 following MCAO. *n* = 6 per group. White indicates the infarct region. (D, E) Neurological tests were conducted to assess neurological deficits in mice treated with MLT, Mф-MLT@FNGs, or vehicle at 1, 3, and 7 days following MCAO. *n* = 6 per group. Data are presented as mean ± SD. *P < 0.05, **P < 0.01, ***P < 0.001, ****P < 0.0001 (one-way analysis of variance followed by Tukey’s *post hoc* test for C; two-way analysis of variance with multiple comparisons followed by Bonferroni’s *post hoc* correction for D and E). All experiments were conducted in triplicate. FNGs: Fluorescent nanogels; MCAO: middle cerebral artery occlusion; MLT: melatonin; mNSS: modified Neurological Severity Score.

To comprehensively evaluate the impacts of melatonin or Mф-MLT@FNGs on brain injury, we used TTC staining to measure infarct lesion volumes on day 3 post-MCAO (**Figure 6A**). Notably, MCAO + MLT mice exhibited a significant reduction in cerebral infarct volume compared with MCAO + Vehicle mice, with no significant infarct observed in Sham + Vehicle mice (**Figure 6B** and **C**). Compared with melatonin, Mф-MLT@FNGs further reduced the infarct lesion. Next, we performed mNSS and corner turning tests in mice on days 1, 3, and 7 post-MCAO to assess neurological deficits. The mNSS scores and ipsilateral turning (%) of the MCAO + Vehicle group were markedly increased compared with those in the Sham + Vehicle group (*P* < 0.05), indicating pronounced neurological impairment (**Figure 6D** and **E**). Compared with MCAO + Vehicle mice, administration of melatonin in the MCAO + MLT group significantly mitigated this impairment (*P* < 0.05). Notably, the MCAO + Mф-MLT@FNGs group demonstrated a significantly greater reduction in neurological assessment compared to melatonin. These findings strongly indicated that melatonin alleviated ischemic brain damage. Furthermore, compared with melatonin, Mф-MLT@FNGs further reduced the infarct lesion and amelioration of neurological deficits, demonstrating enhanced efficacy. Overall, our results underscored the neuroprotective effects of melatonin and highlighted the potential therapeutic benefits of Mф-MLT@FNGs in ischemic stroke.

### Knockout of microglial cyclic GMP-AMP synthase diminishes the protective effects of melatonin

**Figure 7:**
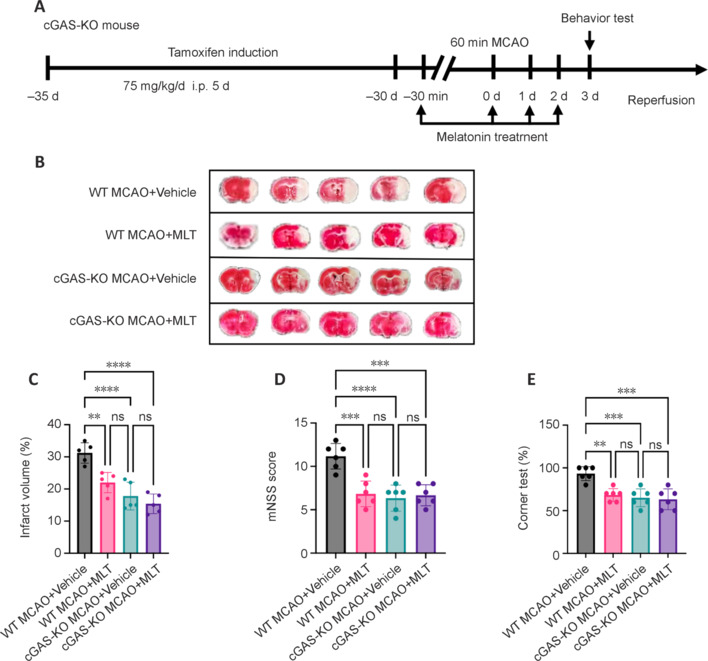
cGAS contributes to the protective effect of melatonin following MCAO. (A) Flowchart depicting the melatonin administration regimen in cGAS-KO and WT mice following MCAO induction. (B, C) Images of 2,3,5-triphenyltetrazolium chloride staining and corresponding quantitative analyses of infarct volume in WT and cGAS-KO mice treated with MLT or vehicle on day 3 following MCAO. *n* = 5 per group. White indicates the infarct region. (D, E) Summarized results of the mNSS and corner test in groups of mice receiving the indicated treatments at 3 days after MCAO. *n* = 6 per group. Data are presented as the mean ± SD. *P < 0.05, **P < 0.01, ***P < 0.001, ****P < 0.0001 (one-way analysis of variance followed by Tukey’s *post hoc* test). All experiments were conducted in triplicate. cGAS: Cyclic GMP-AMP synthase; KO: knockout; MCAO: middle cerebral artery occlusion; MLT: melatonin; ns: not significant; WT: wild-type.

To elucidate the role of microglial cGAS in mediating the neuroprotective effects of melatonin following MCAO, we utilized double transgenic mice with microglia-specific cGAS knockout. These mice were subjected to MCAO and treated with or without melatonin to investigate whether melatonin functions via the cGAS pathway *in vivo* (**Figure 7A**). Our findings revealed that melatonin treatment reduced MCAO-induced cerebral infarct lesions and improved neurological outcomes in WT mice, but these protective effects were abolished in cGAS-KO mice (**Figure 7B–E**). This observation suggested the involvement of cGAS in mediating the beneficial effects of melatonin following MCAO.

## Discussion

This study provides the first evidence that melatonin mitigates ischemic brain injury by targeting a crucial molecule, cGAS, which may regulate the detrimental effects of central microglial pyroptosis and the activation of peripheral immune cells, including neutrophils and monocytes, during the acute phase. This suppressive effect on cGAS was accompanied by reduced expression of inflammatory factors, such as IL-6 and IL-1β, in microglia. Notably, the therapeutic benefits of melatonin in MCAO mice required signaling by the cGAS-STING-NF-κB/NLRP3 inflammasome and NF-κB/GSDMD. These findings highlight melatonin as a potential therapeutic approach for curtailing neuroinflammation and minimizing brain injury following MCAO. Additionally, we developed macrophage membrane-biomimetic and ROS-responsive nanoparticles co-loaded with melatonin (Mф-MLT@FNGs) and explored their neuroprotective advantages in ischemic stroke.

As a pattern recognition receptor, cGAS senses the accumulation of cytosolic dsDNA, initiating an immune response to tissue damage. Inhibition of the cGAS-STING pathway has shown promise in treating ischemic stroke and repairing immune damage (Chauhan and Kaundal, 2023; Yang et al., 2025). Studies have confirmed that melatonin normalizes cGAS-STING signaling by eliminating the excess cytoplasmic DNA induced by cellular stress (Wang et al., 2020; Jauhari et al., 2021). In our study, we observed an increase in DNA damage and the release of dsDNA into the cytoplasm in MCAO mice, as evidenced by elevated levels of γH2AX. Concomitant with the heightened deposition of cytoplasmic dsDNA was upregulation of the cGAS-STING pathway, which melatonin treatment reversed, consistent with prior research. These observations suggest that the cGAS-STING axis may have potential as an indicator of ischemic brain damage during melatonin treatment.

Pyroptosis is a pro-inflammatory form of programmed cell death that relies on the activation of GSDMD in the cytoplasm, driven by factors such as the NLRP3 inflammasome. In this process, active caspase-1 cleaves GSDMD, which then binds to the cell membrane and oligomerizes, forming pores that lead to the release of pro-inflammatory cytokines, such as IL-1β and IL-18 (Ding et al., 2022; Hadian and Stockwell, 2023; Oladapo et al., 2024). Compared with WT mice, microglia-specific cGAS-KO mice showed decreased immunoreactivity of the pyroptosis effector molecules IL-1β, GSDMD, and caspase-1 on the plasma membrane, indicating a role for cGAS in pyroptosis regulation. To assess the therapeutic implications of these findings, we treated MCAO mice with either melatonin or vehicle. Consistent with observations in cGAS KO mice, melatonin treatment improved pyroptosis, as evidenced by immunostaining of the NLRP3 inflammasome and IL-1β along with caspase-1 on western blots. These results indicate that melatonin may reduce the NLRP3 inflammasome, modulate pyroptosis-related proteins, and alleviate the neuroinflammatory burden through inhibition of the cGAS-STING pathway (Ding et al., 2022).

Numerous studies have documented that cGAS-STING activation drives neuroinflammatory processes and plays a crucial role in neurological diseases (Shao et al., 2023). The cGAS-STING pathway functions as an upstream regulator of the NF-κB pathway (Zhong et al., 2024). Pyroptosis is closely linked to the inflammasome, with NF-κB serving as the priming signal for NLRP3, which also participates in the activation of the NLRP3 inflammasome and the subsequent triggering of GSDMD-mediated pyroptosis (Martinon et al., 2009). Additionally, in line with a previous study, our ChIP-assay confirmed NF-κB as a positive transcription regulator of the key pyroptosis executor, GSDMD (Shi et al., 2015; Bedoui et al., 2020; Broz et al., 2020). Furthermore, we observed abundant post-MCAO expression of GSDMD, co-localizing with microglia in the peri-ischemic area. This suggested that the microglia were undergoing pyroptosis, which was effectively inhibited by melatonin. Overall, our findings indicate that melatonin effectively suppresses microglial pyroptosis through NLRP3 inflammasome activation and NF-κB/GSDMD-mediated pyroptosis.

In addition to the post-ischemic stroke activation of resident microglia in the central nervous system, the pores formed by GSDMD in the microglial membrane lead to the release of pro-inflammatory cytokines (e.g., IL-1β and IL-18), which may recruit peripheral immune cells into the brain and activate the central immune microenvironment, exacerbating the deterioration in brain function (Jian et al., 2019). Our examination of these cellular components revealed that melatonin-treated mice exhibited decreased infiltration of peripheral immune cells and a dampened immune response to stroke compared with untreated mice. Specifically, there were reductions in the counts of neutrophils and macrophages, along with decreased microglial production of pro-inflammatory cytokines IL-6 and TNF-α. These findings suggest that melatonin treatment might be capable of simultaneously and synergistically regulating central microglial activation and peripheral immune cell infiltration. In the ACTION I and ACTION II clinical trials, immune intervention with natalizumab (an α4-integrin monoclonal antibody) in acute ischemic stroke did not yield significant clinical efficacy. Natalizumab primarily mitigates neuroinflammation-related brain damage by impeding the migration of peripheral immune cells into the brain; however, it fails to address inflammatory processes occurring within the brain, such as the activation of microglia and the release of danger signals (Elkins et al., 2017; Elkind et al., 2020). In this study, melatonin attenuated the central inflammatory response and reduced the influx of peripheral immune cells into the brain, highlighting its dual mechanism in protecting against secondary ischemic brain injury.

Furthermore, our study demonstrated that melatonin treatment resulted in reduced cerebral infarct volume and significantly improved neurological deficits in MCAO mice. These post-MCAO effects were abolished in cGAS-KO mice, indicating the involvement of cGAS in the protective effects mediated by melatonin. Such results are consistent with previous studies showing that inhibition of the cGAS-STING pathway abrogates the beneficial effects of melatonin on mitophagy, cell survival, and cardiac function (Wang et al., 2020; Jauhari et al., 2021). Despite these promising effects, melatonin exhibits rapid degradation and a short half-life *in vivo* (Harpsøe et al., 2015), which may limit its sustained efficacy in the inflammatory microenvironment following cerebral ischemia. Nanomaterials have been widely explored in biomedical research, including ischemic stroke (Moujalled et al., 2021), with PLGA-based nanoparticles showing considerable promise in both treatment and diagnosis (Yan et al., 2023; Lv et al., 2024; Ma et al., 2024). PLGA, a US Food and Drug Administration-approved polymer, safely degrades into carbon dioxide and water in the body, making it biocompatible and nearly non-toxic (Zhi et al., 2021). To extend the half-life and enhance the bioavailability of melatonin, we employed PLGA-based nanoparticles as a delivery vehicle. Previous studies have highlighted macrophages as key cellular effectors in inflammation and tissue repair, infiltrating ischemic brain regions and sites of inflammation shortly after the onset of cerebral ischemia. The ability of macrophages to effectively modulate the inflammatory microenvironment has made macrophage membrane camouflage a promising approach for nanoparticle delivery to ischemic lesions (Li et al., 2021; Ma et al., 2024). Notably, integrins α4 and β1, which are highly expressed on macrophage surfaces, interact with vascular cell adhesion molecule-1 on the endothelium at ischemic sites, promoting macrophage migration to ischemic brain tissue (Li et al., 2021). Thus we utilized a macrophage membrane camouflage strategy to enhance the stability and targeting efficiency of melatonin delivery via Mф-MLT@FNGs. Indeed, our research revealed that Mф-MLT@FNGs showed enhanced melatonin-related benefits. Thus, by addressing the rapid degradation limitation while offering targeted delivery to ischemic brain tissue, Mф-MLT@FNGs provide a promising option for improving the therapeutic efficacy of melatonin in ischemic stroke.

This study has some limitations. First, while the use of cGAS-KO mice provided valuable insights, we did not include a sham control group to account for baseline conditions, moreover, the lack of cGAS re-expression limits causality confirmation. Future studies should employ rescue experiments, such as transgenic re-expression of cGAS in cGAS-KO mice, to determine whether the restoration of cGAS impacts the neuroprotective effects observed with melatonin (e.g., infarct reduction, neurological improvement, and pyroptosis suppression). Second, the study did not examine the long-term safety and effects of the Mф-MLT@FNG nanoparticles, which may affect their clinical applicability and should be investigated in future research.

In conclusion, the present study provides insights into the complex interplay between melatonin and the immune response and highlights a potential neuroprotective mechanism of melatonin through modulation of the cGAS pathway in ischemic stroke. Activation of the cGAS-STING pathway amplifies neuroinflammation by activating central microglial pyroptosis and recruiting peripheral inflammatory cells to the ischemic brain through cytokine (IL-1β) secretion, ultimately exacerbating brain injury. Our findings offer new insights into the inflammatory mechanisms underlying ischemic stroke and suggest potential therapeutic targets and strategies for improving stroke outcomes.

## Additional files:

***Additional Figure 1:*** *Predicted binding sites for nuclear factor kappa-B in the gasdermin D promoter by JASPAR.*

Additional Figure 1 Predicted binding sites for nuclear factor kappa-B in the gasdermin D promoter by JASPAR.
JASPAR: A Database of Transcription Factor Binding Profiles, https://jaspar.genereg.net/). Promoter sequences were retrieved, and motif enrichment analysis was conducted using the JASPAR CORE collection.

***Additional Figure 2:*** *Ultraviolet absorption features and in vitro release of melatonin from MLT@FNGs in the presence of 0.5 mM*H_*2*_*O*_*2*_*.*

Additional Figure 2 Ultraviolet absorption features and in vitro release of melatonin from MLT@FNGs in the presence of 0.5 mM H_2_O_2_.
(A) The full-spectrum ultraviolet absorption of MLT@FNGs was measured in the presence and absence of H_2_O_2_. (B) Drug release curve showing the relationship between the percentage of drug release (calculated as the ratio of the amount of drug released at a given time to the total drug content) and time. FNGs: Fluorescent nanogels; MLT: melatonin.

## Data Availability

*All relevant data are within the paper and its Additional files*.
