# Supplementary figures and images for: Melatonin alleviates neuroinflammation in ischemic stroke by regulating cyclic GMP-AMP synthase– mediated microglial pyroptosis signaling
Source: Neural Regen Res. 2025 Jun 19;21(6):2380–8. doi: 10.4103/NRR.NRR-D-24-01070 (PMC13211821; doi:10.4103/NRR.NRR-D-24-01070)

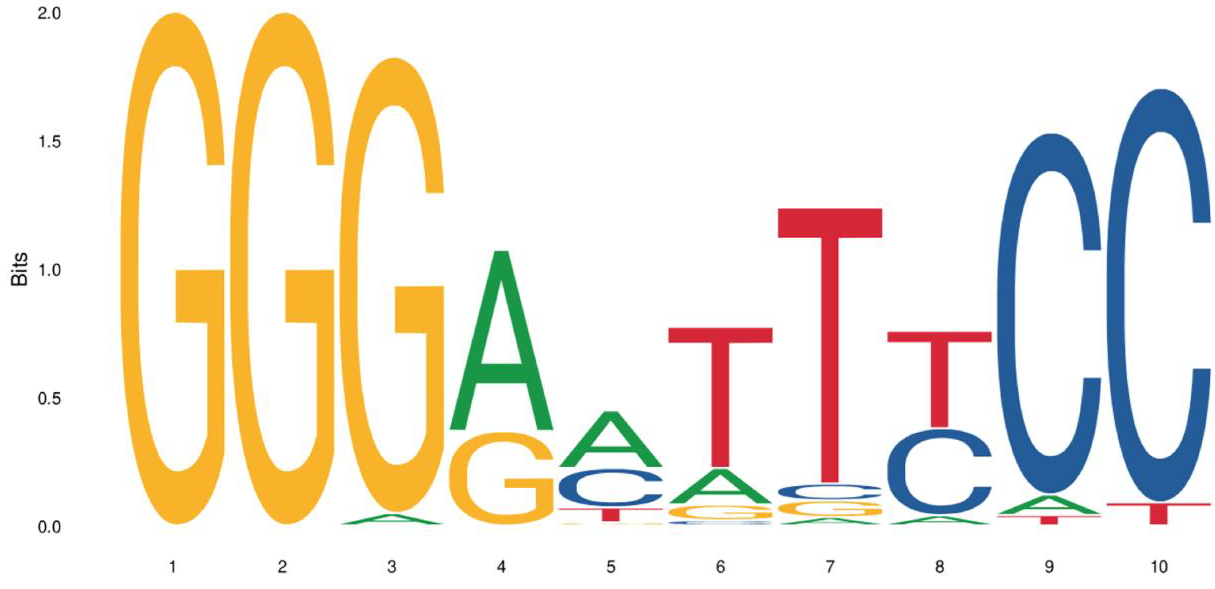

Supplement: Supplementary file 1 [file NRR-21-2380_Suppl1.tif]

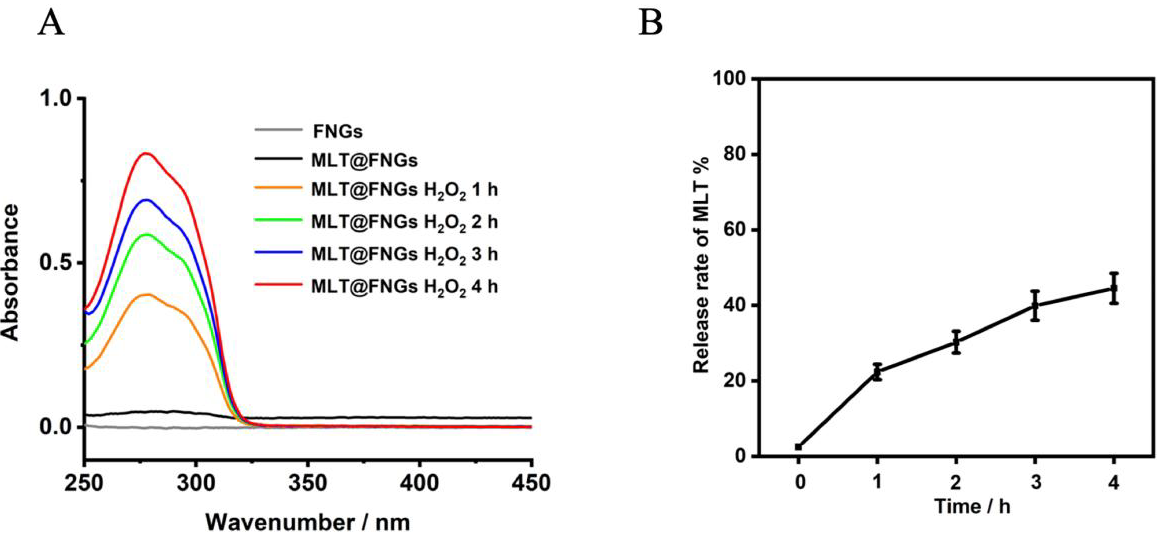

Supplement: Supplementary file 2 [file NRR-21-2380_Suppl2.tif]
